# Early Outcome of a Contemporary Unicondylar Knee System

**DOI:** 10.7759/cureus.74596

**Published:** 2024-11-27

**Authors:** Kevin B Fricka, Alexander V Strait, Henry Ho, Robert H Hopper, Craig J McAsey

**Affiliations:** 1 Orthopaedics, Anderson Orthopaedic Clinic, Arlington, USA; 2 Adult Hip and Knee Replacement, Anderson Orthopaedic Research Institute, Alexandria, USA

**Keywords:** journey ii unicondylar knee, kaplan-meier survivorship, koos jr, patient-reported outcome measures, promis global health mental, promis global health physical, reasons for revision

## Abstract

Introduction: In 2019, a new fixed-bearing implant for unicondylar knee arthroplasty (UKA) was introduced that incorporated a round-on-flat design featuring an Oxinium femoral component coupled with a highly crosslinked polyethylene bearing surface. Compared to prior implants, the design featured smaller size increments coupled with medial and lateral-specific tibial baseplates. The objective of this study is to describe our institution’s early experience with this UKA implant system by evaluating survivorship, reasons for revision, and patient-reported outcome measures (PROMs).

Methods: The 944 UKAs that comprise the study population, including 814 medial and 130 lateral joint replacements, were performed by six surgeons from September 2019 through the end of December 2023. All UKA components were cemented, the mean age at surgery was 67.1±10.0 years, 57% (537/944) of the UKAs were performed among women, and the mean body mass index was 30.3±6.2 kg/m^2^. Outcome measures included Kaplan-Meier survivorship using reoperation for any reason as an endpoint, reasons for reoperation, range of motion, and PROMs that were collected preoperatively and postoperatively at 4 weeks, 4 months, 1 year, and 2 years.

Results: Using reoperation for any reason as an endpoint, 1-year survivorship was 99.3% (95% CI, 98.7 to 99.9%) and 2-year survivorship was 98.2% (95% CI, 96.9 to 99.5%). There were nine reoperations among the 944 UKAs, including three for infection, two for tibial loosening, one for femoral loosening, one for progression of arthritis, one for patellar instability, and one for recurrent hemarthrosis. The mean time to reoperation was 0.82±0.68 years and all were performed among medial UKAs. Range of motion increased from 117±8 degrees preoperatively to 122±6 degrees at 1-year follow-up. PROMs, including the Knee Injury and Osteoarthritis Outcome Score for Joint Replacement (KOOS JR) and the Patient-Reported Outcomes Measurement Information System (PROMIS) Global Health Physical score, demonstrated progressive increases from the preoperative assessment through 2-year follow-up. For UKAs with preoperative and 2-year KOOS JR scores, the mean change was 31±17.

Conclusion: As new implants and technologies are introduced, documenting the early outcome from high volume centers can offer valuable insights to validate the effectiveness of implant systems before they are adopted by the broader orthopedic community. This study demonstrates encouraging early outcome data associated with a contemporary UKA system based on survivorship and PROMs. At this early stage, there are no implant-related concerns but obtaining additional follow-up will be important to quantify how revisions and PROMs evolve over time.

## Introduction

Due to its less invasive nature compared to total knee arthroplasty (TKA), unicondylar knee arthroplasty (UKA) is an appealing treatment option for patients with single-compartment degenerative changes who have failed more conservative management. Proposed benefits of UKA include faster initial recovery [1,2], improved functional outcomes [3-7], the ability to return to sporting activities [7-9], and kinematics that are more similar to the native knee [2,10]. Historically, the primary disadvantages of UKA have been higher rates of revision and decreased implant survival compared to TKA [2,11]. However, registry data and a large meta-analysis indicate that surgeons who perform the largest proportion of UKAs in their practice have the lowest revision rates and achieve an average 10-year survival of 94% [12,13]. Still, multiple national joint registries demonstrate that UKAs continue to comprise a relatively small proportion of all knee arthroplasties performed, with current percentages ranging from 4 to 10% [11,14,15].

With the benefit of several decades of clinical experience, the development of contemporary UKA implants often focuses on improving upon prior designs that have achieved the best long-term clinical outcomes. In 2019, a new fixed-bearing UKA was introduced (Journey II UK Unicondylar Knee System or JUK, Smith & Nephew, Memphis, Tennessee) with the goal of optimizing the implant for the largest possible patient population by offering smaller size increments coupled with medial and lateral-specific tibial baseplates. The design, which incorporates a round-on-flat geometry featuring an Oxinium femoral component coupled with a highly crosslinked polyethylene bearing surface, represents an evolution of a prior implant system (Zimmer Unicompartmental High Flex Knee or ZUK, Warsaw, Indiana). Among fixed-bearing UKA designs, the ZUK has yielded some of the lowest cumulative revision rates (between 5 and 6% at 7-year follow-up) in multiple national registries [16,17]. In addition to implant design changes, the JUK system also incorporates updated instrumentation, including a novel tibial cutting guide with improved ability to recut, if needed, and the flexibility to shift the femoral component to minimize edge loading before committing to a final femoral position.

Owing to the large number of options currently available, the process of selecting the optimal implant for a particular patient can be a daunting undertaking for both the patient and the surgeon. New systems are intended to improve on prior designs but their clinical performance is unknown. In order to better inform the decision-making process related to implant selection, it is important to share early outcome data related to new implants and emerging technologies. The objective of this study is to describe our institution’s early experience with the JUK by evaluating survivorship, reasons for revision, and patient-reported outcome measures (PROMs).

## Materials and methods

After an Institutional Review Board determined that this study met the requirements for exemption, a prospectively maintained database was queried for all primary UKAs performed at our institution using JUK components. The UKAs included in this study were performed from September 2019 through to the end of December 2023. Primary UKAs that did not use JUK components were excluded from the study population. The query identified 944 UKAs performed by six surgeons that included 814 medial and 130 lateral joint replacements. All JUK components were cemented, the mean age at surgery was 67.1±10.0 years, 57% (537/944) of the UKAs were performed among women, and the mean body mass index was 30.3±6.2 kg/m^2^. The 944 JUKs included 112 patients with staged bilateral UKAs that had been performed between September 2019 and December 2023 with a mean of 283±305 days between the first and second procedures.

The JUK surgical technique involves cutting the tibia first with the aid of a stylus or reference spoon that can be placed between the proximal tibia and femur to determine resection depth. At our institution, the medial procedure was performed through a medial incision extending from just superior to the patella to the tibial tubercle utilizing either a midvastus or medial parapatellar approach. The lateral procedure was performed utilizing a lateral skin incision and a lateral parapatellar arthrotomy. After the tibial resection was performed using a tibial jig that requires only a single pin for fixation, spacer blocks ranging from 8 to 14 mm were used to determine joint balance in extension and flexion. In the event an 8 mm spacer block did not fit, the tibial system includes a recut mechanism that allows the guide to be reattached and dropped to cut an additional 1-2 mm without adding any additional pins for fixation. Gap balancing was evaluated and adjusted (Table 1) with the goal of achieving an extension gap balance with 1-2 mm of laxity and a flexion gap balance with 2-3 mm of laxity.

**Table 1:** Gap Balancing Strategy for UKA with the JUK UKA: Unicondylar Knee Arthroplasty; JUK: Journey II UK Unicondylar Knee System

Status of Flexion Gap	Status of Extension Gap		
	Tight in extension	Balanced in extension	Loose in extension
Tight in flexion	Recut 1-2 mm of tibia	Use osteotome to remove 1-2 mm of posterior femoral cartilage or add tibial slope.	Resect 1 mm less distal femur and either add more tibial slope or resect 1-2 mm posterior femoral cartilage.
Balanced in flexion	Resect 1-2 mm of distal femur	Good balance - Proceed with the case	Resect 1-2 mm less distal femur
Loose in flexion	Resect 1-2 mm of distal femur and increase poly thickness	Shift femoral component 1 mm posterior to resect less posterior condyle	Increase poly thickness by 1-2 mm

**Figure 1:**
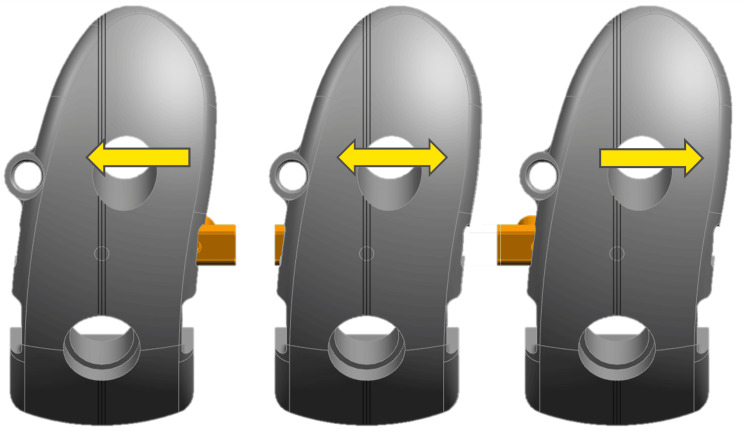
JUK Femoral Trial Adjustment Using reference lines on the femur and tibia, the femoral trial can be shifted 1-3 mm medial or lateral to achieve optimal position and tracking before the final lug holes are drilled. JUK: Journey II UK Unicondylar Knee System

The appropriate spacer block was subsequently used to set the distal femoral cutting guide. The distal femoral cut was completed and gap balancing was reconfirmed. The femoral component was then sized so it would be just shy of the cartilage tidemark on the medial side and 1-2 mm below the tidemark on the lateral side. The femoral component lug positions are combined in three groupings that include sizes 1-3, 4-7, and 8-10 which allow for fine-tuning the optimal femoral size as needed. Once the femoral resections were complete, the surgeon trialed the appropriate size tibia and polyethylene thickness. Using reference lines on the femur and tibia, the femoral trial was shifted 1-3 mm medial or lateral (Figure 1) to achieve optimal position, avoid edge loading, and optimize tracking before the final lug holes were drilled.

Once the desired position had been achieved, the femoral and tibial preparations were completed, and the trials were removed. Since the JUK is a cement-only system, cement was mixed and pressurized into the bone after multiple small holes had been drilled into the femoral and tibial surfaces to help with cement penetration. Care was taken to remove any residual cement after polymerization had occurred and the final polyethylene liner was locked into place.

The primary outcome measure for this report is survivorship using reoperation for any reason as an endpoint. Secondary outcomes included reasons for reoperations, range of motion (ROM), and PROMs including the Knee Injury and Osteoarthritis Outcome Score for Joint Replacement (KOOS JR) as well as the Patient-Reported Outcomes Measurement Information System (PROMIS) Global Health Physical and Mental scores. Outcome data were collected preoperatively (defined as within 180 days of surgery) and postoperatively at 4 weeks (defined as 21 to 35 days after surgery), 4 months (defined as 99 to 141 days after surgery), 1 year (defined as 0.75 to 1.25 years after surgery), and 2 years (defined as 1.75 to 2.25 years after surgery). No radiographic analysis was performed in conjunction with this study. At the time the data for this analysis was compiled, 370 UKAs had follow-ups that were obtained at least 1.75 years after surgery. The mean follow-up for all 944 UKAs was 1.28±1.00 years.

Categorical data are summarized using frequencies while continuous data are reported using the mean ± standard deviation. Data normality was evaluated using a Kolmogorov-Smirnov test. Comparisons between two independent groups comprised of normally-distributed data were performed using an independent samples t-test while three or more groups were compared with an ANOVA. A Mann-Whitney test was used to compare two groups with nonparametric distributions and a Kruskal-Wallis test was used for three or more groups. For paired data, a paired samples t-test was performed for normally distributed data, and a Wilcoxon signed-rank test was used for nonparametric distributions. Correlations between continuous variables were evaluated using Pearson’s correlation for parametric data and Spearman’s correlation for nonparametric data. Survivorship was evaluated using the Kaplan-Meier method with reoperation for any reason as an endpoint. The threshold for statistical significance was defined as 0.05. Data analyses were performed using IBM SPSS Statistics for Windows, v28.0 (IBM Corp, Armonk, NY). The Anderson Orthopaedic Research Institute received funding from Smith & Nephew to support the conduct of this study.

## Results

**Figure 2:**
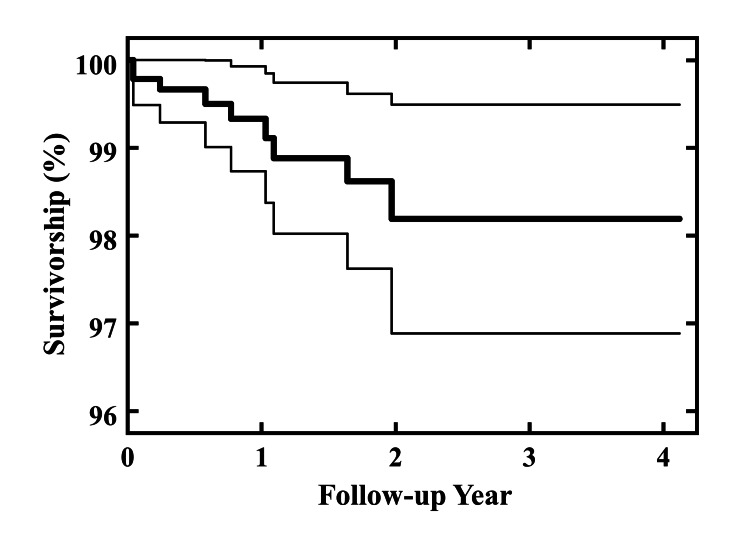
Kaplan-Meier Survivorship Using Reoperation for Any Reason as an Endpoint The bold line corresponds to the survivorship and the thinner lines designate the 95% confidence intervals.

Using reoperation for any reason as an endpoint, 1-year survivorship was 99.3% (95% CI 98.7 to 99.9%) and 2-year survivorship was 98.2% (95% CI, 96.9 to 99.5%, Figure 2).

There were nine reoperations among the 944 UKAs included in the study population including three for infection, two for tibial loosening, one for femoral loosening, one for progression of lateral compartment arthritis, one for patellar instability, and one for recurrent hemarthrosis (Table 2).

**Table 2:** Reoperations TKA: total knee arthroplasty

Time to Reoperation	Reason for Reoperation	Components Revised
13 days	Infection	Insert only
15 days	Infection	Insert only
89 days	Patellar instability	None
213 days	Tibial loosening	All with conversion to TKA
282 days	Progression of lateral compartment osteoarthritis	All with conversion to TKA
1.03 years	Recurrent hemarthrosis	Insert only
1.09 years	Tibial loosening	All with conversion to TKA
1.64 years	Femoral loosening	All with conversion to TKA
1.97 years	Infection	All with single-stage conversion to TKA

Five of the reoperations were conversions to a TKA, three involved exchanges of the polyethylene insert only, and the reoperation for patellar instability reconstructed the medial patellofemoral ligament without revising any of the primary components. For all nine UKAs, the time to reoperation was 0.82±0.68 years and all occurred among medial UKAs. Among cases with data, ROM increased from 117±8 degrees preoperatively (N=673) to 122±6 degrees at 1-year follow-up (N=226). Among 202 cases with preoperative and 1-year data, ROM increased from 118±8 degrees to 122±7 degrees (p<0.001).

**Figure 3:**
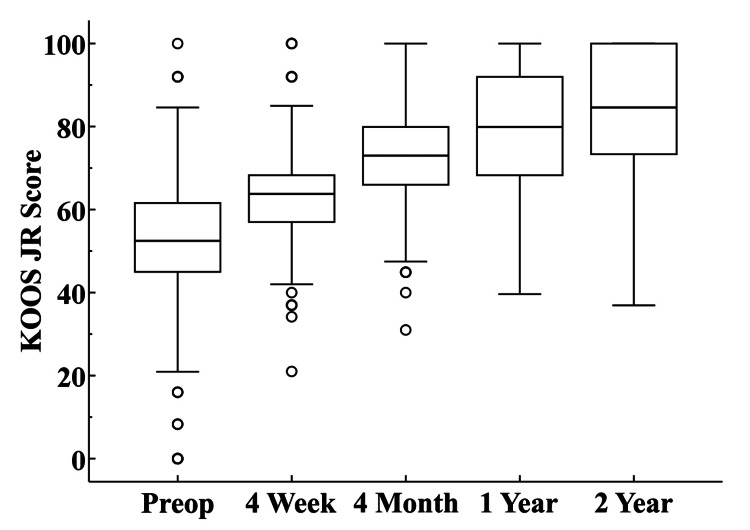
KOOS JR Scores The box at each interval represents the interquartile range and the circles designate outlier values that are more than 1.5 times above or below the interquartile range. The horizontal line within each box illustrates the median value. KOOS: Knee Injury and Osteoarthritis Outcome Score

**Figure 4:**
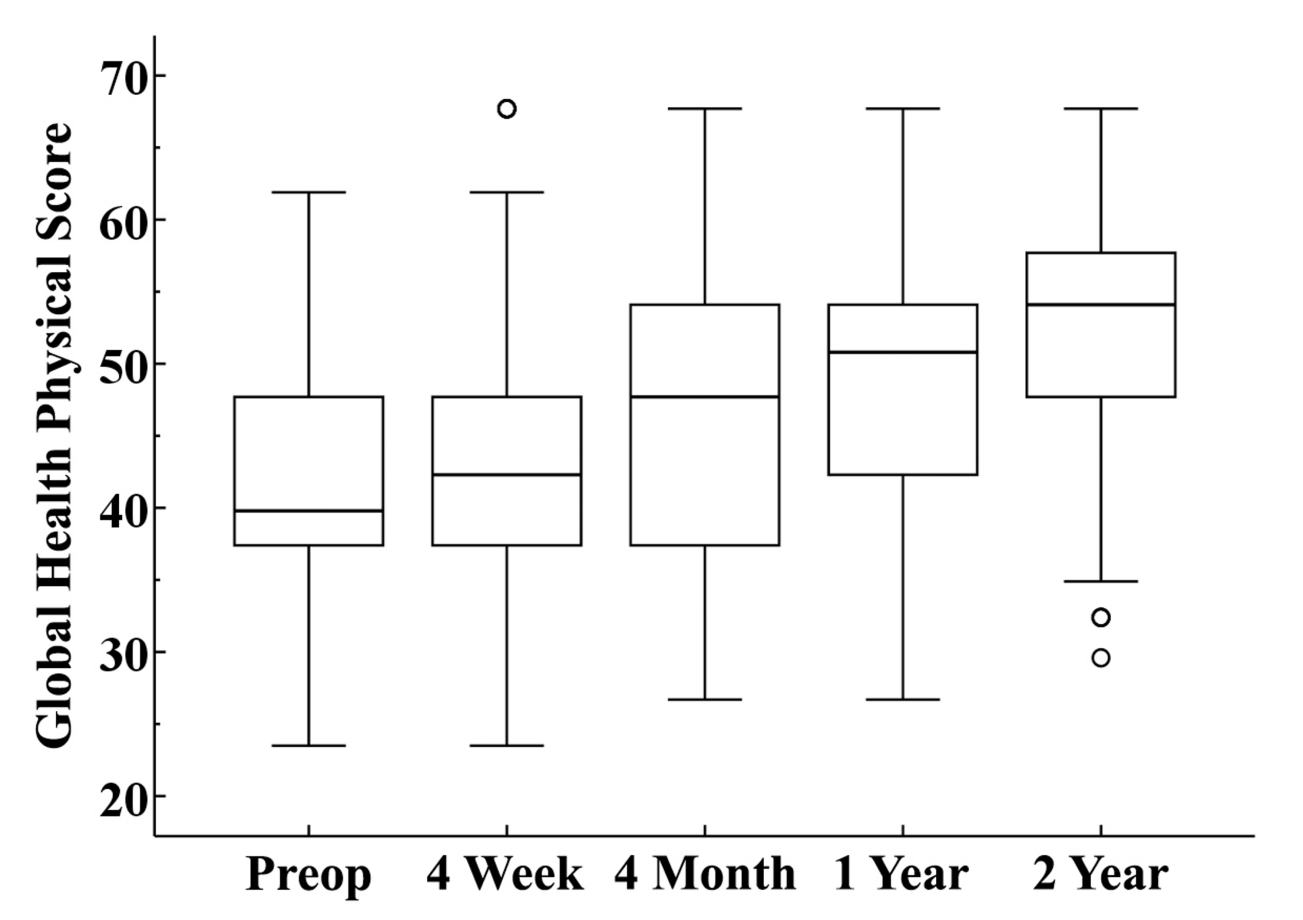
Global Health Physical Scores The box at each interval represents the interquartile range and the circles designate outlier values that are more than 1.5 times above or below the interquartile range. The horizontal line within each box illustrates the median value.

**Figure 5:**
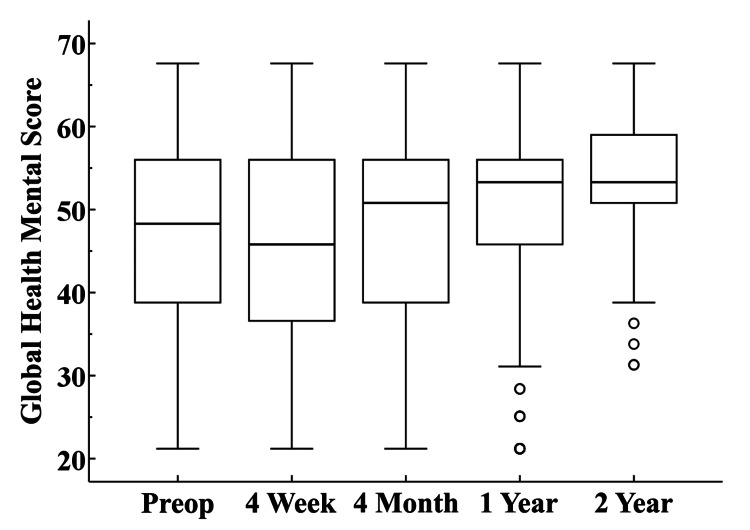
Global Health Mental Scores The box at each interval represents the interquartile range and the circles designate outlier values that are more than 1.5 times above or below the interquartile range. The horizontal line within each box illustrates the median value.

PROMs tended to demonstrate progressive increases from preoperative through 2-year follow-up (Table 3, Figures 3-5).

**Table 3:** Patient-Reported Outcome Scores Std Dev: Standard Deviation, N: Number of unicondylar knee arthroplasty cases; PROMIS: Patient-Reported Outcomes Measurement Information System; KOOS: Knee Injury and Osteoarthritis Outcome Score

Interval	KOOS JR				PROMIS Global Health Physical				PROMIS Global Health Mental			
	Mean	Std Dev	Median	N	Mean	Std Dev	Median	N	Mean	Std Dev	Median	N
Preop	53	14	52	728	42	8	40	762	47	12	48	769
4-Week	63	10	64	635	43	8	42	645	46	12	46	650
4-Month	73	13	73	512	46	9	48	509	47	12	51	515
1-Year	79	15	80	373	49	9	51	382	51	11	53	384
2-Year	83	15	85	285	52	7	54	266	54	8	53	268

**Figure 6:**
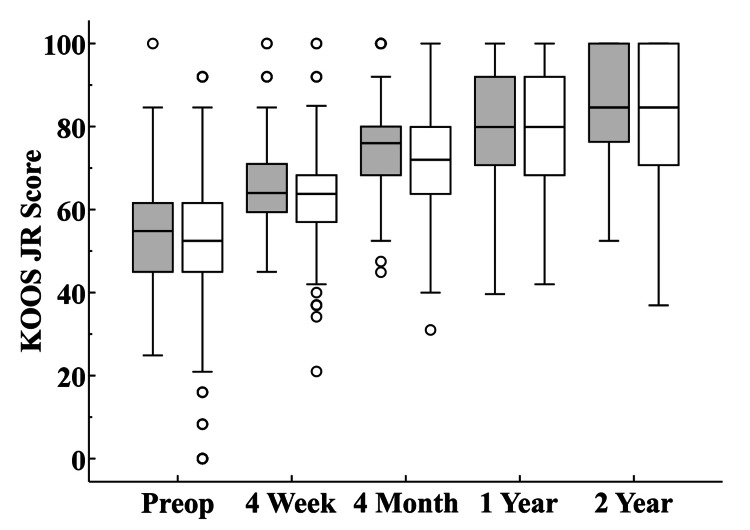
KOOS JR Scores Stratified by Data Completeness The 81 knees with KOOS JR data at all intervals are designated by the boxplots shaded gray while the boxplots for knees with incomplete data are unshaded. The boxes at each interval represent the interquartile range and the circles designate outlier values that are more than 1.5 times above or below the interquartile range. The horizontal line within each box illustrates the median value. KOOS: Knee Injury and Osteoarthritis Outcome Score

Although the KOOS JR scores for the 81 UKAs with data at all intervals (preoperative, 4-week, 4-month, 1-year, and 2 years) tended to be higher at the 4-week (p=0.02) and 4-month (p=0.02) follow-up intervals compared to those that did not have complete KOOS JR data, the magnitude of the differences was modest (Figure 6).

There was no statistical difference among the KOOS JR scores for the 81 knees with data at all intervals compared to those that did not have complete KOOS JR data at the preoperative (p=0.94), 1-year (p=0.27), or 2-year (p=0.11) intervals (Figure 6). Based on the number of cases available, there were also no differences in the age at surgery (p=0.17) or body mass index (p=0.18) among knees with KOOS JR scores at all intervals, those with scores at some intervals, and those without any scores (Table 4).

**Table 4:** Patient Demographics Stratified by KOOS JR Completeness UKAs with KOOS JR scores at the preoperative, 4-week, 4-month, 1-year, and 2-year intervals are classified as having Complete KOOS JR Score Data, UKAs missing scores at any interval are classified as Incomplete, and UKAs without any KOOS JR scores are classified as None. Std Dev: Standard deviation; KOOS: Knee injury and Osteoarthritis Outcome Score

Demographic	Type of KOOS JR Score Data									p-value
	Complete			Incomplete			None			
Number of UKAs	81			803			60			N/A
Parameter	Mean	Std Dev	Median	Mean	Std Dev	Median	Mean	Std Dev	Median	
Age at Surgery (years)	68.4	9.3	69.3	67.1	9.9	68.0	65.1	10.8	64.2	0.17
Body Mass Index (kg/m^2^)	29.1	5.4	28.1	30.4	6.4	29.4	30.8	5.7	29.8	0.18

There was also no difference based on sex with females comprising 56% (45/81) of the cases with complete KOOS JR data, 57% (457/803) of the cases with incomplete data, and 58% (35/60) of the cases with no KOOS JR data (p=0.95). For knees with preoperative and 2-year KOOS JR scores, the mean change was 31±17 and 84% (194/232) met or exceeded the Minimum Clinically Important Difference (MCID) of 14 proposed by Lyman et al. [18]. At the 2-year follow-up, there was a positive correlation between the KOOS JR and PROMIS Global Health Physical score (Spearman correlation coefficient=0.54, p<0.001) that was similar to the strength of the correlation between the PROMIS Global Health Physical and Mental scores (Spearman correlation coefficient=0.57, p<0.001). The correlation between the KOOS JR and the PROMIS Global Health Mental score at the 2-year follow-up was positive but weaker (Spearman correlation coefficient=0.24, p<0.001).

## Discussion

As new implants and technologies are introduced, documenting the early outcome from high volume centers can offer valuable insights to validate the effectiveness of implant systems before they are adopted by the broader orthopedic community. This multi-surgeon study found 1-year survivorship to be 99.3% and 2-year survivorship was 98.2%. Implant-specific early outcome data from high volume institutions is particularly important in the absence of national registry data. At the time that this manuscript was prepared, the 2023 annual reports represented the most recent publicly available national registry data. In our review of major national registries, we were unable to find outcome data specific to the JUK. A review of the literature identified only one study that described the early outcome of the JUK and it reported no complications or revisions among 145 medial UKAs with minimum 2-year follow-up [19]. Our current study and the findings of D’Amario et al. [19] compare favorably with the results of other UKA and TKA designs reported in multiple national registries [11,14,15].

Several limitations of the current study should be considered. Although data are prospectively entered into our institutional database, this study was retrospective and lacks a control group. While all patients who have an email address on file receive reminders about completing online follow-up PROMs, not all patients choose to respond. To characterize our contemporary experience, all UKAs performed using JUK implants from the time they were first used at our institution through the end of 2023 were included in our analysis. Because the patients in our study population had a range of follow-up durations, we elected not to impose a minimum follow-up duration and employed survivorship to characterize implant performance which also enabled us to quantify the uncertainty associated with incomplete follow-up. It is possible that some patients may have elected to seek treatment elsewhere, so we may not be aware of all reoperations. The relatively short-term follow-up interval may mitigate this concern since patients are likely inclined to return to their primary surgeon when they experience problems not long after their initial procedure. Since not all patients elected to respond to surveys, we compared those UKAs with KOOS JR scores at all follow-up intervals with those that did not have complete data. Although we cannot know the outcome of the patients who never responded, the absence of difference between UKAs with KOOS JR scores at all follow-up intervals and those missing some scores is reassuring. The absence of differences in the demographics among patients who completed KOOS JR questionnaires at all intervals, those who completed surveys at some intervals, and those who did not complete any surveys suggest that the patients who responded to surveys are likely representative of the entire study population. Since this study did not incorporate a radiographic analysis, including this data in future reports would be useful, particularly when longer follow-up is available. Because the surgeons who performed the UKAs in our study population are high-volume joint replacement specialists and it has been shown that higher volume UKA surgeons have better outcomes than those who perform fewer UKAs [20], our findings may not be generalizable to other institutions, particularly those where UKA is not frequently performed. Similarly, the relatively low revision rate in our study compared to the UKA data in national registries, which include outcomes from low UKA volume surgeons, could be due, in part, to our surgeons’ volume and their experience with UKA.

Although the total number of reoperations was limited, the reasons for reoperation in the current analysis are similar to those reported in prior studies. Infection was the most frequent reason for reoperation in our population while it was the second most common cause in the Swedish registry and the fourth most common cause in the Australian registry [15,21]. The second most common cause of reoperation in this study was loosening which was also the second most common cause in the Australian registry [15]. In both the Australian and Swedish registries, the progression of disease was the most common reason for revision [15, 21] so it will be important to continue evaluating the outcome of the JUK to assess how the reasons for revision evolve over time.

A unique aspect of this study is the inclusion of PROMs obtained shortly after surgery. The PROMs evaluated for this study tended to show progressive improvement through 2-year follow-up. The exception was the Global Health Mental score at 4-week follow-up which demonstrated a slight decrease relative to the preoperative score. By 4-month follow-up, the Global Health Mental score was similar to the preoperative score, and progressive improvement was observed at 1 and 2-year follow-up. These PROM trends may be useful when surgeons counsel their patients about what to expect in the short term as they recover from their UKA surgery.

Although lateral UKAs are performed less frequently than medial UKAs, our study population included 130 lateral UKAs and there were no reoperations among these cases and no lateral tibial baseplate failures in this study. The JUK is the only currently available system with a lateral-specific tibial baseplate and the anatomic design is intended to allow for better fit of the implant which may lead to improved cement fixation. The anatomical design may also facilitate improved positioning of the tibia on the lateral side and avoid overhang/underhang issues associated with non-anatomic lateral tibial baseplates.

## Conclusions

This study demonstrates encouraging early outcome data associated with the JUK system based on survivorship and PROMs. At this early stage, there were no wear-related concerns, lending support to the contention that the JUK design and surgical technique optimize femoral and tibial position to minimize any edge loading that could lead to premature polyethylene wear. The JUK system technique combined with the Oxinium femoral component and the use of highly crosslinked polyethylene should also minimize any wear-related complications in the long term. The KOOS JR and PROMIS Global Health scores tended to demonstrate progressive improvement through 2-year follow-up which may help surgeons as they counsel their patients about what to expect following UKA. Based on currently available data, the surgeons at our institution continue to use the JUK for medial and lateral UKA. Obtaining additional follow-up will be important to quantify how revisions and PROMs evolve over time. Our current data indicates that the JUK UKA system performs well in the short term and should give surgeons confidence when performing medial and lateral partial knee replacements in their patients.
